# Evolution of dominance in gene expression pattern associated with phenotypic robustness

**DOI:** 10.1186/s12862-021-01841-6

**Published:** 2021-06-06

**Authors:** Kenji Okubo, Kunihiko Kaneko

**Affiliations:** 1 Department of Basic Science, Graduate School of Arts and Sciences, University of Tokyo, Komaba 3-8-1, Tokyo, 153-8902 Japan; 2 Center for Complex Systems Research, Universal Biology Institute, University of Tokyo, Tokyo, Japan

**Keywords:** Mendelian dominance, Gene regulatory network, Robustness, Diploid, Evolution, Theoretical model

## Abstract

**Background:**

Mendelian inheritance is a fundamental law of genetics. When we consider two genomes in a diploid cell, a heterozygote’s phenotype is dominated by a particular homozygote according to the law of dominance. Classical Mendelian dominance is concerned with which proteins are dominant, and is usually based on simple genotype–phenotype relationship in which one gene regulates one phenotype. However, in reality, some interactions between genes can exist, resulting in deviations from Mendelian dominance. Whether and how Mendelian dominance is generalized to the phenotypes of gene expression determined by gene regulatory networks (GRNs) remains elusive.

**Results:**

Here, by using the numerical evolution of diploid GRNs, we discuss whether the dominance of phenotype evolves beyond the classical Mendelian case of one-to-one genotype–phenotype relationship. We examine whether complex genotype–phenotype relationship can achieve Mendelian dominance at the expression level by a pair of haplotypes through the evolution of the GRN with interacting genes. This dominance is defined via a pair of haplotypes that differ from each other but have a common phenotype given by the expression of target genes. We numerically evolve the GRN model for a diploid case, in which two GRN matrices are added to give gene expression dynamics and simulate evolution with meiosis and recombination. Our results reveal that group Mendelian dominance evolves even under complex genotype–phenotype relationship. Calculating the degree of dominance shows that it increases through the evolution, correlating closely with the decrease in phenotypic fluctuations and the increase in robustness to initial noise. We also demonstrate that the dominance of gene expression patterns evolves concurrently. This evolution of group Mendelian dominance and pattern dominance is associated with phenotypic robustness against meiosis-induced genome mixing, whereas sexual recombination arising from the mixing of genomes from the parents further enhances dominance and robustness. Due to this dominance, the robustness to genetic differences increases, while optimal fitness is sustained to a significant difference between the two genomes.

**Conclusion:**

Group Mendelian dominance and gene-expression pattern dominance are achieved associated with the increase in phenotypic robustness to noise.

**Supplementary Information:**

The online version contains supplementary material available at 10.1186/s12862-021-01841-6.

## Background

Mendelian inheritance is a keystone of genetics. Mendel’s law [1] concerns binary traits and consists of three laws: the law of gene segregation, the law of independent assortment, and the law of dominance.

First, the law of segregation is explained by meiosis from a modern viewpoint. The law is mainly discussed with regards to the genetic segregation of alleles, whereas segregation of phenotypes is not intensively studied.

Second, the law of independent assortment is explained by the “independent” expression of different genes [2]. Mendel’s law of independence has been reinterpreted regarding the independence of genes and the independence of traits. Originally, Mendel conjectured independent segregation of genes, but later, the dependence of segregation was known to occur frequently. Traits can be independent when determined by genes that segregate independently. Such independence, however, does not always hold as will be discussed below.

Third, Mendel’s law of dominance assumes a pair of alleles of a gene for the same locus, which involves a nonlinear interaction between them. A binary trait (phenotype) is given by A and a. If the two alleles at the given locus are homozygote and *AA* (*aa*), the trait is A (a). For a heterozygote, i.e., *Aa*, the trait is A if A is dominant. Indeed, by creating a pure line of a genotype with *AA* and *aa*, Mendel showed that F1, a hybrid of *AA* or *aa*, expresses only the dominant character A, whereas the next generation from F1 shows the character A or a according to a 3:1 ratio. In the classical Mendelian law, the phenotype of concern is which protein from different alleles exists or functions dominantly.

In reality, there are many genes at different loci. Nevertheless, their independence is assumed in the classical Mendelian inheritance. Even if there are many genes, when the expression is independent, each trait is determined by the corresponding gene according to simple genotype–phenotype relationship [3]. However, if several genes contribute to each trait as in complex and quantitative traits, it is unlikely that they will be independent. Note that correlations between traits can arise because the genetic content is correlated, as in linkage disequilibrium. Furthermore, “genetic interactions” often exist between genes because gene products work together to produce phenotypes.

In general, multiple genes at different loci often interact with each other, resulting in complex genotype–phenotype relationship. Recent studies have demonstrated that genes often constitute a gene regulatory network (GRN). Although it is possible to establish a pure line for the genes that correspond to a specific trait, other “hidden genes” at different loci may influence the trait. Consequently, the law of dominance can be violated, with the traits of F2 deviating from the Mendelian ratio of 3:1, as is measured by the interaction deviation [4, 5]. Furthermore, even in the homozygous case, the phenotype can be perturbed by noise [6, 7]. This can also result in a deviation from the Mendelian ratio.

In contrast, the possibility of generalizing Mendelian dominance beyond the simple single-gene case has been discussed previously. Fisher posited that Mendelian dominance could arise because of evolutionary benefits concerning robustness [8]. Alternatively, Wright argued that metabolic stability could lead to dominance [9]. However, to date, both arguments remain hypothetical, and any quantitative relationship linking the robustness in gene expression or metabolic dynamics to Mendelian dominance remains elusive.

In general, the degree of deviation and robustness depend on the nature of gene expression dynamics governed by the GRN, shaped through evolution. Indeed, in the haploid case, extensive studies have been conducted on the nature of gene expression dynamics governed by gene–gene interactions under the influence of stochasticity. The evolution of genotype–phenotype relationship has been investigated numerically using the GRN model, thus elucidating the evolution of robustness to noise and mutations [10–13].

In fact, recent studies have revealed that the regulatory interactions (promoters, transcription factor binding sites, and enhancers) change through evolution more often than each gene that codes a specific protein itself. Hence, the evolution of GRN with rewriting of the network has been extensively investigated. Now, it is essential to extend these studies of gene expression dynamics to the diploid case [14, 15]. For it, we consider gene expression dynamics as a result of two GRNs in two genomes in diploid organisms, and we seek a possibility that this dominance is extended to the gene expression levels as phenotypes.

Recall that the classical Mendelian dominance concerns which proteins of two alleles are dominant, i.e., which protein from the alleles at the same locus is dominant due to protein–protein interaction or the protein’s function. Here, in contrast, we are interested in gene espression levels determined by GRNs. Depending on the two GRNs as two genomes in diploid organisms, the expression levels of proteins are different. One protein is synthesized from one of the genomes, whereas it is not expressed from the other, and instead, another protein can be expressed. Then, for the protein expression level as phenotype, one may discuss the dominance similarly to in the Mendelian case, even though the two alleles do not determine the phenotype of concern at the same locus. Instead, it is determined collectively as a group of genes constituting a GRN.

There are two possibilities for considering such an extension of dominance. One straightforward way is seeking the possibility of dominance between expression patterns from two genomes in diploid organisms. Let us first compute the gene expression pattern for one group of genes, for homozygotes ${\tilde{A}}{\tilde{A}}$ or ${\tilde{B}}{\tilde{B}}$ with the same genome groups. Next, we compute the gene expression pattern of heterozygotes ${\tilde{A}}{\tilde{B}}$. Then, we examine whether the expression pattern of the heterozygote is biased dominantly toward one of the homozygote expression patterns. If the expression of each gene is independent, such bias would not be expected. In contrast, if genes regulate with each other properly through the GRN, such bias, i.e., the dominance of homozygote gene expression pattern, might exist.

This is a fundamental question, especially considering that quantitative traits are explained by pleiotropic expression and are the results of multiple genes. Hence, in this study, we introduce such expression-pattern dominance as a pattern of genes and numerically examine whether such dominance appears due to the evolution of GRN.

Even though the above pattern dominance could be a theoretically natural measure, it may not be easy to measure experimentally. As a natural extension of single-gene Mendelian dominance, it may be desirable to measure the dominance for the expression of a single gene within a genome. However, in this case, we need to introduce a group of population of genomes because many possible genomes produce the same expression for a given gene. In this study, we introduce group Mendelian dominance (GMD) for a group of population of genomes generated by applying the procedure to make a pure line. Here, each group can share the corresponding expression, even if their GRNs are different. This GMD is in contrast to the classical Mendelian dominance described between alleles.

First, we study both the pattern dominance and GMD, whereas we pay more attention to the latter, as it can be comparable with the Mendelian dominance. Here, by simulating the evolution of diploid GRNs, we seek to determine whether the degree of GMD evolves beyond the classical Mendelian dominance for each gene. Specifically, we examine whether complex genotype–phenotype relationship can achieve GMD through the evolution of the GRN with interacting genes. We adopt a computational model of the GRN and adapt it to the diploid case, noting that proteins can be transcribed from the two genes in the diploid case. We introduce a mode of inheritance to account for sexual recombination and meiosis in the diploid cell, where the expression dynamics result from the superposition of two GRNs through meiosis segregation, sexual recombination, and mutation. Next, the results of the evolution simulation demonstrate the evolution of GMD via groups of interacting genes in the GRN. The condition for the evolution of GMD and its possible relationship with robustness to noise and mutation is explored.

Then, we define dominance of gene expression pattern as collective behavior of genes and demonstrate that such dominance also evolves. Additionally, the importance of meiosis-based genome mixing in establishing this dominance is revealed. Finally, we discuss the possible connection of GMD to experimental observations.

## Results

### Evolution simulation based on a diploid GRN model

First, we introduce the theoretical model of the GRN. Here, we consider the gene expression level $x_i$ ($-1<x_i<1$) for the genes $i=1$, ..., *k*. Each gene $i(=1, 2, ..., N)$ has an expression level $x_i(t)$ at time step *t*. In the model, $x_i$ is scaled such that it takes a value between 0 (non-expression) and 1 (expression). Each gene interacts with others and itself, with the interaction of the *j*th gene with the *i*th gene described by the matrix $J_{ij}$ [3, 11, 12, 16, 17]. $J_{ij}$ can take three values: $+1$, $-1$, and 0, which represent the activation of gene *i* by gene *j*, the corresponding inhibition, and no interaction, respectively.

We adopt a discrete-time model [3, 6, 18, 19], in which the expression level $x_i(t+1)$ in the next time step determined by $\sum _{j=1}^{N}J_{ij}x_j(t)$ represents the GRN. A small Gaussian noise, $N(0,\sigma )$, is added to the dynamics to account for stochasticity in the gene expression. By using the sigmoid function $f[x] = 1/(1+\exp [-\beta x])$ with large $\beta (=100)$, the expression dynamics are given by

1 $$\begin{aligned} x_i(t+1)=f\left[ \sum _{j=1}^N J_{ij}(x_j(t)-\theta ) \right] + \sqrt{x}\eta (0, \sigma ), \end{aligned}$$

which is the discrete-time version of the continuous-time model for gene expression dynamics [10, 17], where $\theta$ is the threshold for the expression level. The initial expression levels $\{x_i(0)\}$ take a value of 0 for *i* from 1 to $N-1$ and 1 for *i* in *N*. These $\{x_i(0)\}$ are fixed for all individuals over generations. After a certain time *T*, $x_i(t)$ reaches a fixed point for most cases, where $x_i$ is 0 or 1 depending on the structure of the network $J_{ij}$. Thus, after sufficient time steps (*T*), the expression pattern $x_i(t)$ determines the phenotype. Note that there are some cases in which the expression pattern $x_i(t)$ cannot reach a stable state; however, these are rare.

Diploid cells have two genomes that correspond to two matrices, $J^{(1)}_{ij}$ and $J^{(2)}_{ij}$ [14, 15, 19, 20]. The gene expression dynamics of diploid GRN are determined by the sum of the products from two genomes such that the dynamics are given by modifying $f[\sum _{j=1}^N J_{ij}(x_j(t)-\theta )]$ to $\frac{1}{2}(f[\sum _{j=1}^{N} J^{(1)}_{ij}(x_j(t)-\theta )] + f[\sum _{j=1}^{N} J^{(2)}_{ij}(x_j(t)-\theta )])$:

2 $$\begin{aligned} x_i(t+1) =\frac{1}{2}\left( f\left[ \sum _{j=1}^{N} J^{(1)}_{ij}(x_j(t)-\theta )\right] + f\left[ \sum _{j=1}^{N} J^{(2)}_{ij}(x_j(t)-\theta )\right] \right) + \sqrt{x}\eta (0, \sigma ). \end{aligned}$$

The threshold $\theta$ is set at 0.5 unless otherwise mentioned. This threshold value makes the states 0 and 1 symmetric. Here, we mainly study this case as we intend to investigate the Mendelian dominance without prior bias between the dominant and recessive states. Genetic changes take three main forms: asexual, meiosis only (i.e., without recombination), and the standard sexual mode, including meiosis and recombination. This paper defines these as asexual, meiosis only (MO), and meiosis and recombination (MR). In the asexual mode, the two genomes are not mixed, i.e., $J^{(1)}_{ij}$ and $J^{(2)}_{ij}$ are copied. The other two modes involve sexual reproduction. In MO, one genome from parent-1 becomes a new $J^{(1)}_{ij}$ (gamete) and one genome from parent-2 becomes a new $J^{(2)}_{ij}$. In MR, two genomes from parent-1 (parent-2) are mixed by recombination to provide a gamete, that is, a new $J^{(1)}_{ij}$ ($J^{(2)}_{ij}$). In recombination, $J^{(1)}_{ij}$ and $J^{(2)}_{ij}$ are mixed by a row vector in both parents to produce a gamete. Recall that mutation is included as a change in the matrix element $J_{ij}$ with 0 or ±1 in all three modes. A fourth mode, involving recombination only (RO), is included as a reference to elucidate whether meiosis or recombination is more critical for GMD (see "Methods" for details). MO and MR correspond to the case in which a single pair of homologous chromosomes is used in diploid cells. On the other hand, asexual and RO correspond to the case with a single chromosome.

Here, fitness is defined by how closely the expression pattern $\{x_i\}$ matches a prescribed target. After the above modes and mutation, those with higher fitness are selected for the next generation. (See "Methods" for details of the selection procedure.)

### Evolution of fitness

**Fig. 1:**
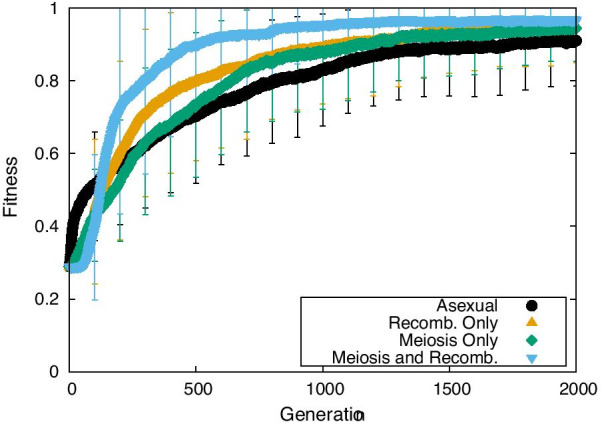
Fitness as a function of evolutional generation for the asexual (black line), MR (blue line), and MO (green line) modes. The mutation rate per edge = $4\times 10^{-4}$ and the strength of noise = 0. The increase in fitness was saturated within 2000 generations. The average of over 50 samples is plotted. The error bar represents the standard deviation (SD) over them

The simulated evolution of the fitness is shown in Fig. 1, with the evolution of the average fitness of the population plotted for the asexual, MO, and MR modes. In each case, fitness increased beyond 90% of the maximum, within 500–1000 generations (Fig. 1), with MR and MO exhibiting the fastest and slowest increase, respectively.

**Fig. 2:**
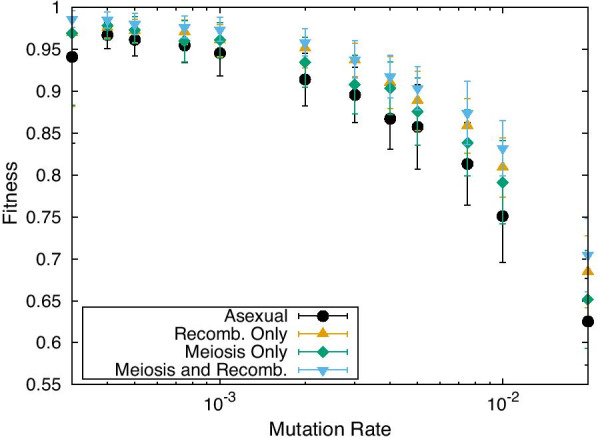
Mutation rate dependence of the average fitness. The average fitness was computed for 100 individuals representing the last generation in the evolution of over 10,000 generations for the asexual (black circles), MR (blue triangles), and MO (green diamonds) modes. The noise amplitude $\sigma$ was 0.0. For increasing mutation rates, error catastrophe occurs, which prevents the average fitness from rising through evolution over 10,000 generations. The recombination-only reference case is also plotted (orange triangles). The average over 50 samples is plotted. The error bar represents the SD over them

Figure 2 shows the mutation rate dependence of the fitness. For each mode, as the mutation rate increases, the increase in fitness stops. A drop begins at a mutation rate of approximately $10^{-3}$. For MR, this decline is slightly suppressed and is initiated at a slightly higher mutation rate. This decline is caused by the loss of information at higher fitness values owing to the accumulation of mutations in the network, as discussed in the context of error catastrophe [21]. Although the recombination process in MR slightly suppresses this error catastrophe, the effect is not significant.

### Group Mendelian dominance

Next, we investigated whether and how GMD evolves. As mentioned in the Background, the inter-gene interactions result in deviation from the 3:1 ratio of Mendelian dominance [4, 5]. Here, we rename the phenotypes $+1$ and 0 with p and m with normal font. The corresponding genomes are written in italics, i.e., $p_1$ and $m_1$.

We create a pure line for a homozygous case in which two genomes have the same $J_{ij}$ [22]. There are a variety of matrices (genes) that produce p (m) for a specific $x_i$, which are given by $p_1p_1, p_2p_2, p_3p_3$, ..., $p_{n^p} p_{n^p}$ ($m_1m_1, m_2m_2, m_3m_3$, ..., $m_{n^{m}}m_{n^{m}}$). From the p (${p_1, p_2}$, ..., $p_{n^{p}}$) and m (${m_1, m_2}$, ..., $m_{n^{m}}$) sets, we generate $N_{sample}$ homozygotes, *pp* and *mm*, and $2N_{sample}$ heterozygotes, *pm*, the phenotypes of which can be examined. Then, we count the number of each case, denoted by $N^{pp}_{p}$, $N^{pm}_{p}$, and $N^{mm}_{m}$, respectively (here, we can assume that the number of $N^{pm}_{p}$ is larger than $N^{pm}_{m}$ and that p is dominant, by renaming p and m if $N^{pm}_{m}>N^{pm}_{p}$). When Mendel’s law of dominance is perfect, $N^{pp}_{p}=N_{sample}$, $N^{pm}_{p}=2N_{sample}$, and $N^{mm}_{m}=N_{sample}$. However, in general, the genes are not independent: within the groups p and m chosen as a group of genomes that produce each trait, each fraction can deviate from a ratio of 1:2:1. Therefore, we define the group Mendelian ratio (GMR) as the fraction $(N^{pp}_{p}+N^{pm}_{p}+N^{mm}_{m})/4N_{sample}$. The deviation of the GMR from unity corresponds to the interaction deviation in quantitative genetics [4, 5] (see "Methods" for the detailed algorithm).

### Evolution of group Mendelian ratio

**Fig. 3:**
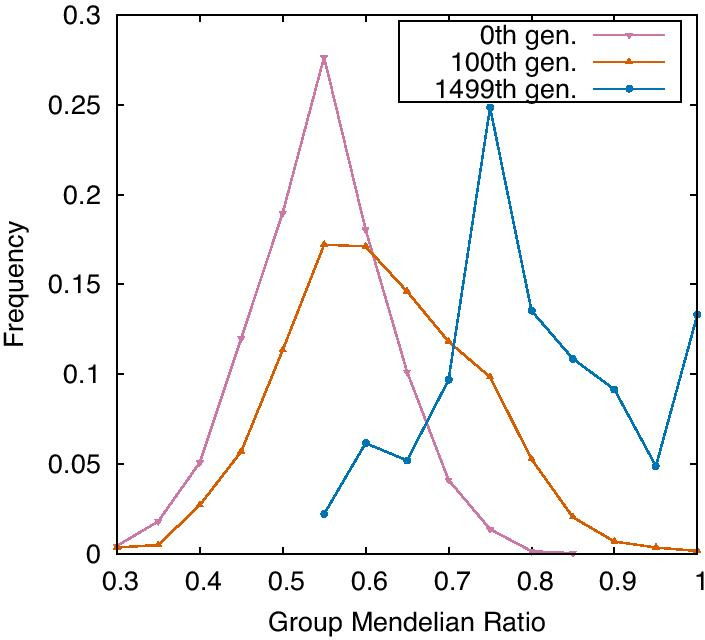
Frequency of group Mendelian ratio (GMR) in the 0th, 100th, and 1499th generation. This frequency is calculated by each focused gene in 30 samples. GMR is increased along the evolution and over 10% of them reach complete GMD. The mutation rate per edge = $10^{-4}$ and the strength of noise = $10^{-4}$. This frequency is measured in MR mode. The bin is 0.05

This GMR increased through evolution. Here, the GMR depends on each gene, and hence, we computed the frequency distribution of GMR over all genes. In Fig. 3, this frequency distribution is plotted for the three generations. In the 0th generation, the peak of GMR frequency is approximately 0.5 because for the random GRN chosen at the 0th generation genes the expression levels are distributed symmetrically for [0,1]. The average and maximum GMRs increase with the evolution. At the 1499th generation, the peak around 0.75 means that their expression levels are always 0 or 1, whereas there is another peak at 1, which implies complete GMD, as will be explained later.

#### Condition for the increase of the group Mendelian ratio

**Fig. 4:**
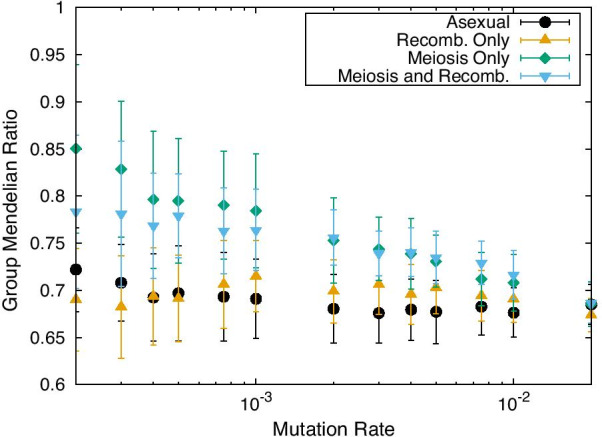
GMR as a function of mutation rate. The GMR at the $10^4$-th generation is shown for the asexual (black dots), MR (blue triangles), and MO (green diamonds) modes. The noise amplitude was 0.0. Data for the RO reference case are also plotted (orange triangles). The average over 50 samples is plotted. The error bar represents the SD over them

We studied the dependence of the GMR on the mutation rate (Fig. 4). In the MO and MR modes, the average value of the GMR almost exceeds 0.75 for mutation rates less than $10^{-3}$ (note that this is an average for all genes *i*: for $x_i$ of some gene GMRs reaching 1). For mutation rates exceeding $10^{-3}$, the average GMR decreased, and the value for MR was higher than that for MO. In contrast, for the asexual case, the GMR remained at a much lower level ($\sim$0.7). Note that, in the case of recombination without meiosis, GMR remained at the same low level as in the asexual case.

Recall that GMR is 0.5 if all the GRNs are random (see "Methods" for details). If the GMR is sufficiently high, GMD at a group level occurs . Specifically, we regard GMD to be achieved when the average GMR in the population is over 0.75. Therefore, GMD is realized for the MO and MR modes, i.e., when meiosis is considered.

### Correlation between group Mendelian ratio and robustness to noise

According to Wright, Mendelian dominance might have some correlation to the robustness of the phenotype shaped by the dynamics. As the present GRN model contains the change in phenotype by noise, we investigated the robustness of the phenotype against noise. Note that as the robustness of the fitness, i.e., its insensitivity, increases, the fitness variance decreases. Hence, we computed the SD of fitness.

#### Phenotypic fluctuation and group Mendelian ratio

**Fig. 5:**
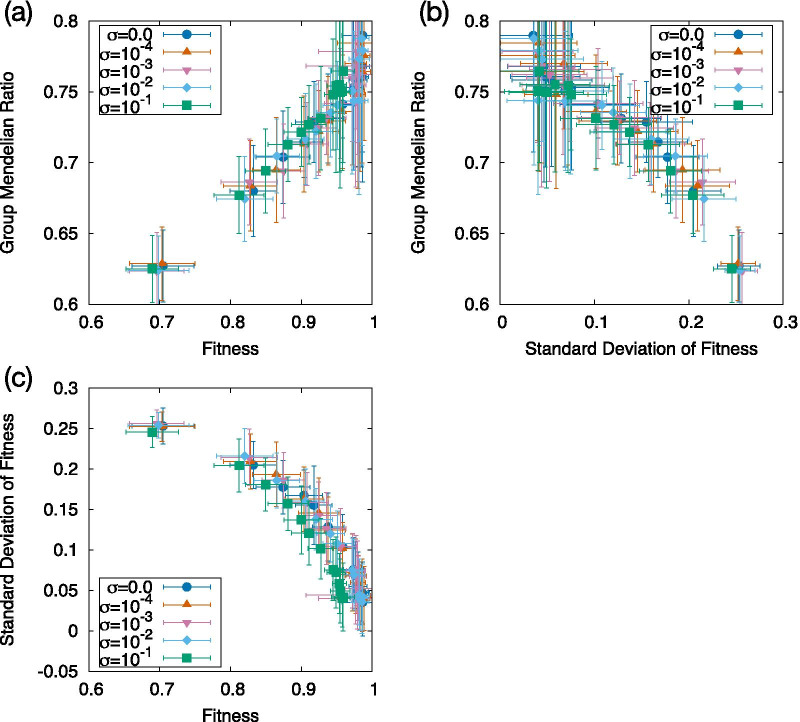
Correlation between the GMR, average fitness, and SD of the fitness. Each variable was computed as the value of the $10^4$-th generation for the MR case. The points were obtained across different mutation rates from $2\times 10^{-4}$ to $2\times 10^{-2}$, as indicated by the color of the data, while the noise level satisfied $0.0\le \sigma \le 0.1$. **a** GMR as a function of the average fitness, **b** GMR as a function of the SD of the fitness, **c** SD of the fitness as a function of the average fitness. Note that **a**and **c**each have three branches corresponding to different noise levels, whereas (**b**) does not, implying that the GMR is dominantly correlated with the fluctuation of the fitness rather than its average. The average over 50 samples is plotted. The error bar represents the SD over them. Correlation coefficient is (**a**) 0.952, **b** $-0.973$, **c** $-0.926$

We have shown that the mutation-rate dependence of the GMR for the MR and MO cases is highly correlated with that of the fitness, as is evident upon comparing Fig. 2 with Fig. 4. Therefore, we plotted the correlation between the GMR and the fitness in Fig. 5a. As shown, the two exhibit a strong correlation. Nevertheless, the plot in Fig. 5a (and Fig. 5c) shows slight deviations depending on the three different noise levels adopted here.

As the noise level varies, the variances of the phenotype and fitness also vary. Therefore, to examine the relationship between the GMR and noise-induced phenotypic variances, we plotted the GMR against the SD of the fitness. In this case, the correlation is more prominent (correlation coefficient = 0.97), with the data not exhibiting noise-level-related branches. All data from different mutation rates and noise levels were well fitted by a single curve, as shown in Fig. 5b. Indeed, the difference between Fig. 5a and b is further explained by the plot of the fitness variance against the average fitness (Fig. 5c), which, again, shows three branches. Together, these three plots indicate that the GMR shows a better correlation with the fitness variance than the fitness itself.

#### Robustness to initial noise and the GMR

**Fig. 6:**
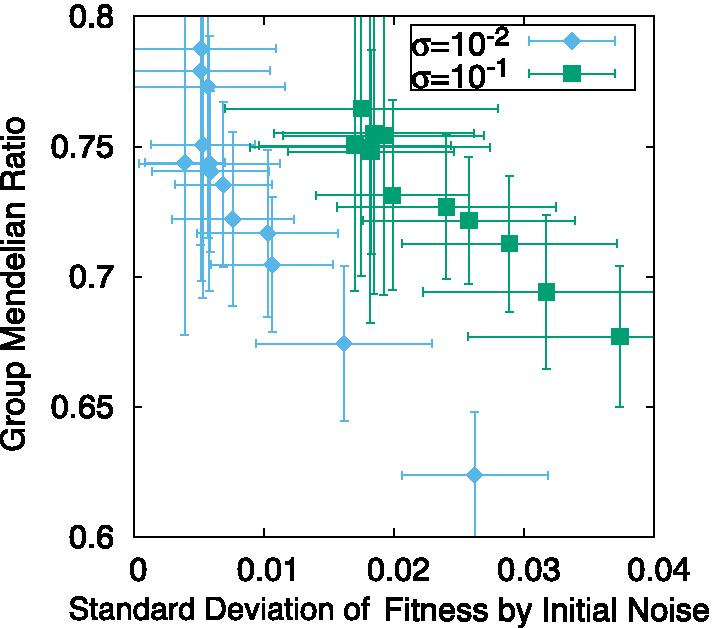
GMR plotted against the isogenetic SD of fitness due to initial noise, i.e., perturbation to the initial conditions. The GMR was computed following the same procedure as in Fig. 5, for the MR case, whereas the fitness variance was computed by distributing the initial condition for $x_i$ with the variance $\sigma$, which is either $10^{-2}$ or $10^{-1}$. The data correspond to the equivalent mutation rates as used in Fig. 5. The average over 50 samples is plotted. The error bar denotes the SD over them

To further confirm the correlation between the GMR and noise robustness, we computed the variance of fitness against the distribution of initial conditions by imposing the noise on $x_i(0)$ (Fig. 6). We computed the SD of fitness against the perturbed initial condition $\{x_i(0)\times (1-\eta (0,0.1))\}$ for a given GRN selected after the evolution, the GMR of which was computed already. The GMR clearly shows a negative correlation with the SD, implying that GMR is positively correlated with the robustness to noise.

#### Group Mendelian ratio and distance between two genomes

**Fig. 7:**
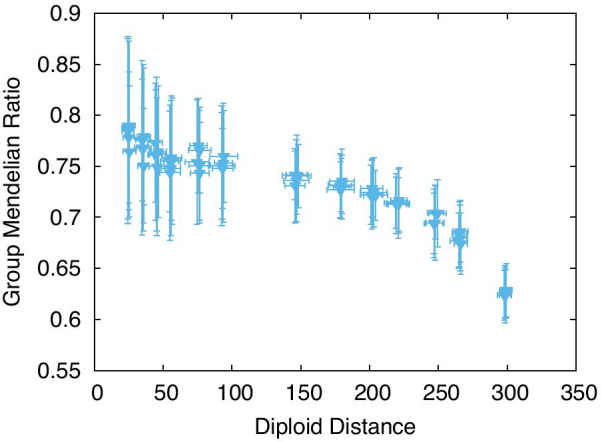
GMR as a function of the distance between the two genomes. The distance was computed as the Euclidean distance between the matrix elements in two genomes, $J^{(1)}_{ij}$ and $J^{(2)}_{ij}$. All data were obtained from the evolved GRNs for the MR case shown in Fig. 5. We simulated GRNs with 20 genes, such that the number of matrix elements, that is, the maximal Hamming distance (i.e., the number of different elements), is $20\times 20=400$. Consequently, the GMR (and the fitness) maintains a high value until half the elements have been changed. The average over 50 samples is plotted. The error bar represents the SD over them

As the phenotype is shaped according to genotype–phenotype relationship by the GRN, it is also essential to study the correlation of the GRN with genetic variance. In diploid cells, the distance between two genomes can provide a measure of genetic variation. Figure 7 shows the relationship between the GMR and the genetic distance between two genomes in a diploid cell. As shown, the GMR begins declining at a certain distance, with the fitness also dropping. Additionally, if the genetic difference between the two genomes is too high, the GMD is not sustained.

In Fig. 7, the GMR can exceed 0.75 when the distance is under 150. Note that the total number of matrix elements (genes) is 20 $\times$ 20, which ensures that the fitness or GMR is maintained, even if over half the elements are different between two genomes. Furthermore, high robustness is achieved to maintain fitness against meiosis. This implies that the fitness of the diploid cell (with meiosis) is remarkably robust against genetic differences between the two genomes. Recall that meiosis mixes genomes in the diploid cells. However, the fitness should be robust to such mixing, which explains the insensitivity of the fitness to meiosis. We added a file of the diploid distance for comparison among the genetic modes (Additional file 1: Fig. S1).

### Pattern dominance

So far, we have studied GMD between a pair of groups of genomes by focusing on the expression of a single gene. As discussed in the Background, another measure for dominance will be between expression patterns over a group of genes that constitute a GRN.

First, from each of the *N* individuals in the evolutionary simulation we extract 2*N* genomes $J^{(n)}_{ij}(n=1...2N)$. For each of the 2*N* genomes, we make a complete copy and create a population of 2*N* complete homozygotes. Then, we compute the expression dynamics in each of the 2*N* complete homozygotes and obtain the expression pattern $x^{{\tilde{n}}{\tilde{n}}}_i$. Next, from the 2*N* genomes, we randomly select two $J^{(l)}_{ij}$, $J^{(m)}_{ij}$, to make a diploid cell consisting of the two heterozygous genomes. Then, we compute the expression dynamics of the heterozygous genomes to obtain the expression pattern of the heterozygote $x^{{\tilde{l}}{\tilde{m}}}_i$.

From the homozygotes ${\tilde{l}}{\tilde{l}}$, ${\tilde{m}}{\tilde{m}}$ and the heterozygote ${\tilde{l}}{\tilde{m}}$, we compute the dominance of the expression pattern from $x^{{\tilde{l}}{\tilde{l}}}_i$, $x^{{\tilde{m}}{\tilde{m}}}_i$ and $x^{{\tilde{l}}{\tilde{m}}}_i$. Here, we compare the expression of such genes that are expressed differently between the two homozygotes: We compute $x^{{\tilde{l}}{\tilde{l}}}_i$ and $x^{{\tilde{m}}{\tilde{m}}}_i$, and choose such genes *i* in which $x^{{\tilde{l}}{\tilde{l}}}_i\ne x^{{\tilde{m}}{\tilde{m}}}_i$. Let the number of *i* that is $x^{{\tilde{l}}{\tilde{l}}}_i\ne x^{{\tilde{m}}{\tilde{m}}}_i$ be $N^{llmm}_{dif}$. Among these *i*, we examine whether the $|x^{{\tilde{l}}{\tilde{l}}}_i-x^{{\tilde{l}}{\tilde{m}}}_i|>|x^{{\tilde{m}}{\tilde{m}}}_i-x^{{\tilde{l}}{\tilde{m}}}_i|$, i.e., the expression of the heterozygote is close to that of the homozygote of *m*, and count the number of such genes, $N^{lm }_{m}$. Because *l* and *m* are arbitrarily chosen numbers, we define the degree of dominance by $\max (N^{lm}_{m}/N^{llmm}_{dif}, N^{lm}_{\ell }/N^{llmm}_{dif})$. If the pattern dominance is complete (i.e., $x^{{\tilde{l}}{\tilde{m}}}_i$ agrees either $x^{{\tilde{l}}{\tilde{l}}}_i$ or $x^{{\tilde{m}}{\tilde{m}}}_i$ in the genes whose expression levels are different between $x^{{\tilde{l}}{\tilde{l}}}_i$ and $x^{{\tilde{m}}{\tilde{m}}}_i$), this degree is closer to 1, whereas if the heterozygote expression pattern matches one parent with half the probability, it takes 1/2. The pattern dominance is larger as this degree approaches 1.

Note that, in the present model, the expression level of each gene $x_i$ is mostly 0 or 1. Then, when there is only a single gene that expresses differently between two homozygotes, the dominance that is computed is always 1, whereas, if the number of differently expressing genes is only 2, the probability of having dominance 1 is 50%, even if the two expressions are totally uncorrelated. To remove such bias, we computed pattern dominance only for pairs of homozygotes $x^{{\tilde{l}}{\tilde{l}}}_i$ and $x^{{\tilde{m}}{\tilde{m}}}_i$, in which more than three genes show different expressions.

**Fig. 8:**
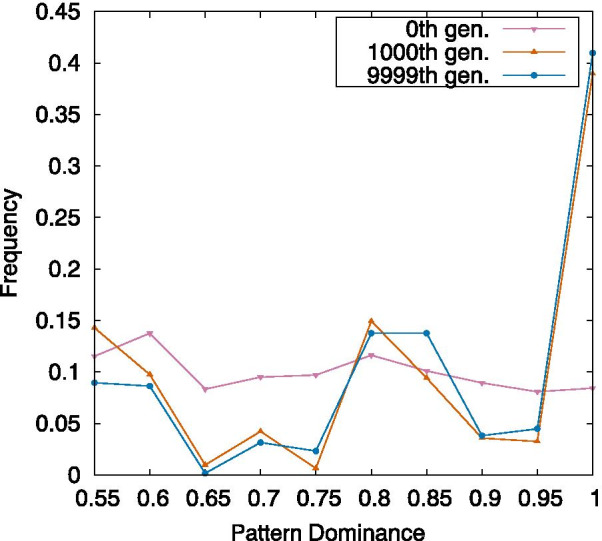
Increase in pattern dominance and evolution. Frequency of pattern dominance in the 0th, 1000th, and 9999th generation. For each generation, this frequency is computed over ten samples. Pattern dominance is increased along with the evolution, and over 50% of heterozygotes reach complete pattern dominance. The mutation rate per edge = $10^{-4}$ and the strength of noise = $10^{-4}$. This frequency is measured in the MR mode. The bin is 0.05

The change in frequency of the pattern dominance computed in this way through evolution is shown in Fig. 8. In generation 0, before evolution, pattern dominance is uniformly distributed between 0.5 and 1. This is because the interaction between the two genomes in GRN heterozygotes is random.

At the 9999th generation, complete dominance is observed for more than 40% of heterozygotes, and approximately 80% of heterozygotes show a dominance value beyond 0.8. This indicates that pattern dominance is evolved.

### Case with distinct protein synthesis from each gene

**Fig. 9:**
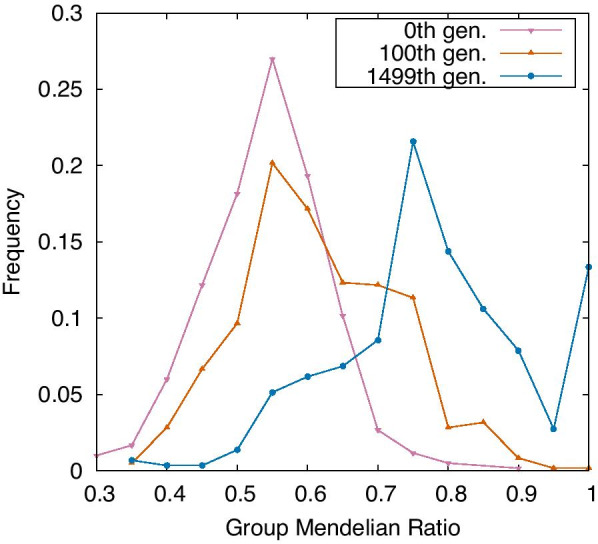
Increase in GMR and the evolution in the case with distinct protein synthesis from each gene. Frequency of GMR in the 0th, 100th, and 1499th generation. This frequency is calculated by each focused gene in 30 samples. GMR is increased along with the evolution, and over 10% of them reached complete GMD. This also supports our results above. The mutation rate per edge = $10^{-4}$ and the strength of noise = $10^{-4}$. This frequency is measured in the MR mode. The bin is 0.05

In this model, $x_i(t)$ is the protein expression (concentration) in a given cell. Once proteins are expressed from either each or both of the genes, one cannot distinguish which of the genes each protein comes from. In contrast, in the classical Mendelian dominance, distinct proteins are synthesized from each gene. Even though the motivation of our study is different, it may also be possible to combine the present study with the classical one. We consider the case in which a certain protein is expressed only from one gene but not from the other. Indeed, such a situation can be included in this model by sorting that $J^{(1)}_{ij}=0$ from $\forall j$. As an example, we carried out the simulation by setting $J^{(1)}_{9j}=0$ for $\forall j$ in genome 1 and $J^{(2)}_{10j}=0$ for $\forall j$. The increase in GMR was observed again (Fig. 9).

### Case with asymmetric model

The model with $\theta =0.5$ is symmetric with respect to 0 and 1. In reality, it is not always symmetric. Hence, we have employed the present model by changing the threshold $\theta$ from 0.5 to 0.3. Our main result (achievement of high GMR and fitness) is not changed by these modifications (Additional file 2: Fig. S2). In the asymmetric model, however, GMR in the 0th generation is already larger than 0.5, so that the degree of increase in GMR is smaller than in the symmetric model because the asymmetry of dynamics causes dominance in the heterozygote and increases GMR.

## Discussion

In this study, we have investigated a diploid GRN model by extending the model described by Omholt et al. [14]. In our model, two GRNs work concurrently to yield the phenotype as a group of gene expressions. By evolving the GRN with mutations, meiosis, and recombination, we have shown that GMD evolves. This dominance is achieved via a pair of groups of various genomes that share a common phenotype determined by the expression of target genes. The degree of dominance increases (corresponding to the decrease of interaction deviation [4, 5]) through evolution, exhibiting a strong correlation with the decrease in phenotypic fluctuations. This evolution of GMD is prominent in the presence of meiosis, which mixes two genomes, whereas, under sexual recombination, the mixing of genomes from the parents further enhances this dominance and robustness. GMD increases the robustness to genetic differences, and the fitness is maintained against significant differences between two genomes.

Indeed, a possible connection between Mendelian dominance and robustness has been posited previously. Fisher discussed the advantages of dominance for the evolutionary robustness of phenotypes [8], while Wright emphasized the relationship between dominance and stability in metabolic networks [9]. However, these discussions were primarily qualitative. Our study has the possibility of extending these qualitative arguments to a quantitative dimension by using gene expression dynamics and a quantitative definition of the GMR.

The GMD at the GRN level in the present study is different from the classical mechanism of Mendelian dominance. In the classical framework, Mendelian dominance is explained by protein–protein interactions and the magnitude of the influence of proteins on the phenotype. However, as shown in this study, the dominance of one phenotype at the expression level can exist at the GRN level. In this case, it is not necessary to consider protein behavior to explain the dominance. Hence, the Mendelian dominance at an individual molecule level can be extended to the pattern level of expressions of many genes that interact with each other through a GRN.

For Mendelian dominance in individuals, the heterozygote advantage has been argued by Goldstein [23] and Poter et al. [24]. Moreover, the relationship between Mendelian dominance and mutational robustness has been investigated by Bagheri, and Wagner [25], whereas the influence of ploidy or recombination on robustness has also been discussed [15, 19, 20].

The degree of group dominance introduced in our model follows the classical method of establishing pure lines and can be measured by proper experiments. For example, Hou et al. [26] tested the degree to which Mendel’s dominance law holds in yeast while also measuring the phenotypic variation. Accordingly, the validity of the negative correlation between Mendelian dominance and the phenotypic fluctuation revealed in our theoretical models can be verified by measuring the degree of dominance in phenotypes and the fluctuations across genes or between different strains, and examining the negative correlation between the two.

We also computed pattern dominance, which is the dominance of the expression patterns of genes that mutually interact through GRN, and found that the pattern dominance is achieved through evolution. If the expression of genes were independent, this would not be expected. The pattern dominance is achieved as a result of interacting genes that evolved to have high fitness. Note that quantitative and complex traits are often formed by the complex interaction of transcribed and translated proteins. The present study suggests that complex quantitative traits may show dominance akin to Mendel’s. In the future, it will be important to explore this possibility beyond the scope of the present study on the evolution of abstract GRN.

For the simulation presented herein, we compared the outcomes of different modes of inheritance, namely asexual, MO, and MR. Our analysis in Fig. 4 concludes that the existence of meiosis is essential to realize GMD. Although the fitness is only slightly changed from in the MR case, the GMR (and genome distance in Additional file 1: Fig. S1) is reduced drastically relative to the MR and MO cases. Therefore, meiosis-induced genome mixing is essential for GMD. Genome mixing between two genomes in the diploid cell requires the GRN to ensure that high fitness is sustained following gene alterations in the other gene. This suggests that the phenotype between two genes in the heterozygote case is identical to that in the “homozygote” case, leading to GMD and pattern dominance as well. From a contemporary viewpoint [2], meiosis is often regarded as consistent with segregation in Mendel’s law. Based on our study, one can conclude that Mendelian dominance stems from Mendelian segregation.

Mendelian dominance in plants has been studied by Huber et al. [27] and Govindaraju [28]. Note that polyploidy is more common in plants. Extending our model to consider ploidy is straightforward, enabling the relationship between dominance and phenotypic fluctuations to be examined and testable predictions to be made. Even though these experimental data have been discussed in the classical Mendelian context, in the future, it may also be essential to look at these and other experimental data from this “extended” Mendelian viewpoint.

## Conclusion

To conclude, we have demonstrated that, through evolution, group Mendelian dominance in a diploid gene regulatory network is achieved at a group level and pattern dominance for the expression over multiple genes interacting with the gene regulatory network. These dominances are evolved to achieve robustness of the fitted state to genetic changes by meiosis and are associated with the increase in phenotypic robustness to noise.

## Methods

### Reasoning of the model

Let us define $y^m_i(t)$ as a concentration of mRNA *i* from each homologous chromosome ($m=1,2$) and $x_i(t)$ as the concentration of protein in a cell. Note that the proteins are synthesized from both of the homologous chromosomes, so that superfix in $x_i(t)$ is not needed. Now the transcription is regulated through GRN, with activation or inhibition from the protein. By replicating the regulation in the diploid cell by $J^{(m)}_{ij}$ $(m=1,2)$ for each gene,

3 $$\begin{aligned} y^{1}_i(t+1) = f\left[ \sum _{j=1}^N J^{(1)}_{ij}(x_j(t)-\theta ) \right] ,\ y^{2}_i(t+1) = f\left[ \sum _{j=1}^N J^{(2)}_{ij}(x_j(t)-\theta )\right] , \end{aligned}$$

where *f*[*x*] is a sigmoid-type function that approaches 1 as *x* is increased and 0 as *x* is decreased, and $\theta$ is the threshold level for the expression. Here, we choose the function $f[x]= 1/(1+\exp [-\beta x])$ specifically. As protein *i* is synthesized from the corresponding mRNA, we obtain

4 $$\begin{aligned} x_i(t+1)= y^1_i(t+1)+y^2_i(t+1)=f\left[ \sum _{j=1}^N J^{(1)}_{ij}x_j(t) \right] + f\left[ \sum _{j=1}^N J^{(2)}_{ij}x_j(t) \right] . \end{aligned}$$

We assume that genes refer to those for which mRNA contributes the construction of the same type of protein even if their mRNA is different. The case in which one of the genes *m* lacks the transcription of mRNA *i* is described by $J^{(m)}_{ij}=0$ for $\forall j$.

### Case with a null allele

For further investigation of $J^{(m)}_{ij}=0$ for the $\forall j$ case above, we have conducted the simulation on the case with a null allele in each gene. In this simulation, one (the other) gene lacks the 9th (10th) gene and is represented by $J^{(1)}_{9j}=0$ ($J^{(1)}_{10j}=0$), respectively.

### Asymmetric dynamics case

When the threshold level is set to $\theta =0.5$, a symmetry exists between the expressed ($x_i=1$) and non-expressed ($x_i=0$) states. We have also investigated the asymmetric type of dynamics, by setting $\theta <0.5$. Specifically, we presented the case with $\theta =0.3$.

### Fitness for evolution

After the expression pattern $x_i(t)$ reaches the stable state $x_i(T)$, the fitness is determined by the distance between the gene expression of the individual and the prescribed target pattern. This target pattern $P_i$ is defined for a part of the genes, namely the “target genes” $i=1, ..., M(<N)$ and each value of $P_i$ is 1 (there is only a single target). $P_i$ is fixed throughout the evolution for all four modes. Here, the phenotype is defined as the time average of $x_i(t)$ for the last ten steps, $\bar{x_i}$. Setting the maximum fitness for $\bar{x_i}-P_i=0$ $(i=1, ..., <M)$, the fitness is defined as $\sum ^{M}_{i=1} |\bar{x_i}-P_i|/2$. According to this fitness landscape, a stable expression pattern $x_i=P_i$ can be more advantageous than an oscillatory state. Then, the selection pressure is set as *S*, such that the probability of obtaining the phenotype $\bar{x_i}$ is proportional to $w=\exp [-S\sum ^{M}_{i=1} |\bar{x_i}-P_i|/2]$.

### Modes of inheritance

Using fitness, a diploid GRN can be produced in the next generation. To introduce genetic variation, we adopted four modes of inheritance: asexual, MO, MR, and RO.

In the asexual mode, offspring are generated as a copy of a parent. One individual, *k*, is chosen as a parent with probability $w_k/W$, where $W=\sum ^L_{k=1} w_k$. The matrices $[J^{(1)}_{ij}]_k$ and $[J^{(2)}_{ij}]_k$ of this parent are copied for the next generation.

In MO, two individuals, $k_1$ and $k_2$, become parents with probabilities $w_{k_1}/W$ and $w_{k_2}/W$, respectively; $k_1$ and $k_2$ must be different. Next, a gamete, $G^{(1)}_{ij}$, is produced from the genome of one parent, $k_1$. One of the two genomes in $k_1$ becomes $G^{(1)}_{ij}$ here. The other gamete, $G^{(2)}_{ij}$, is produced from $k_2$ following the same procedure. These two gametes, $G^{(1)}_{ij}$ and $G^{(2)}_{ij}$, become $J^{(1)}_{ij}$ and $J^{(2)}_{ij}$ for an individual in the next generation.

In MR, the genome mixing process is same as in MO but gametes are generated by a different mode including recombination. Forming a single gamete, $G^{(1)}_{ij}$ ($G^{(2)}_{ij}$), $[J^{(1)}_{ij}]_{k_1}$ ($[J^{(1)}_{ij}]_{k_2}$), and $[J^{(2)}_{ij}]_{k_1}$ ($[J^{(2)}_{ij}]_{k_2}$) are mixed via row vectors with equal probabilities from $k_1$ ($k_2$). In RO, which was investigated as a comparison, there are two parents (parent-1 and parent-2). The *i*th rows of both the $J^{(1)}_{ij}$ and $J^{(2)}_{ij}$ matrices in the next generation are generated from the *i*th row of those from one of the parents. In contrast to MO or MR, this mode does not mix genomes. The RO case would be biologically unrealistic and was added purely to examine the importance of meiosis.

In all four cases, the mutation is added after the offspring are produced. Mutations are implemented by renewing one connection of network $J^{(1)}_{ij}$ or $J^{(2)}_{ij}$ by $+1$, $-1$, or 0 according to the probability $\mu$ per edge (mutation rate).

The evolutionary simulation was conducted using a mutation rate ($\mu$) of $[2\times 10^{-4}$, $2\times 10^{-2}]$, with the SD of noise ($\sigma$) being $[10^{-4}$, $10^{-1}]$. The number of genes (*N*) was set at 20, the ratio of non-zero elements in $J_{ij}$ was not less than 0, the selection pressure *S* was set at 1, and the number of target genes (*M*) was 5. The relaxation time to test the fitness (*T*) was 30.

### Group Mendelian ratio

The GMR was computed by adopting the following procedure. Here, the expression level itself is regarded as phenotype because we consider the situation in which a phenotype is given by the expression of proteins ${x_i}$. Here, we call a single genome from a diploid cell as a haplotype.

1. Making homozygous
  After evolution, the individuals are usually heterozygotes (e.g., ${{\mathcal {J}}_{ij}(1,1){\mathcal {J}}_{ij}(1,2)}$, ${{\mathcal {J}}_{ij}(2,1){\mathcal {J}}_{ij}(2,2)}$, ..., where ${\mathcal {J}}_{ij}(n,m)=J^{(m)}_{ij}$ for the *n*th individual) because it is rare that two genomes are completely identical. Homozygotes are made using the complete copies (e.g., ${{\mathcal {J}}_{ij}(1,1){\mathcal {J}}_{ij}(1,1)}$ and ${{\mathcal {J}}_{ij}(2,1){\mathcal {J}}_{ij}(2,1)}$, ...).
2. Rename haplotypes (Fig. 10)
  The phenotypes $x_i(T)$ in one gene *i* of the homozygote are computed to check whether $x_i\approx 1$ or $x_i\approx 0$. Accordingly, the phenotypes are divided into p or m (e.g., ${{\mathcal {J}}_{ij}(1,1)}{{\mathcal {J}}_{ij}(1,1)}$ expresses the p phenotype). Note that these signs do not indicate dominance or recessiveness. Haplotypes are renamed according to which phenotype is expressed in the homozygote, p or m (e.g., ${{\mathcal {J}}_{ij}(1,1)}$ is renamed ${p_1}$) .

**Fig. 10:**
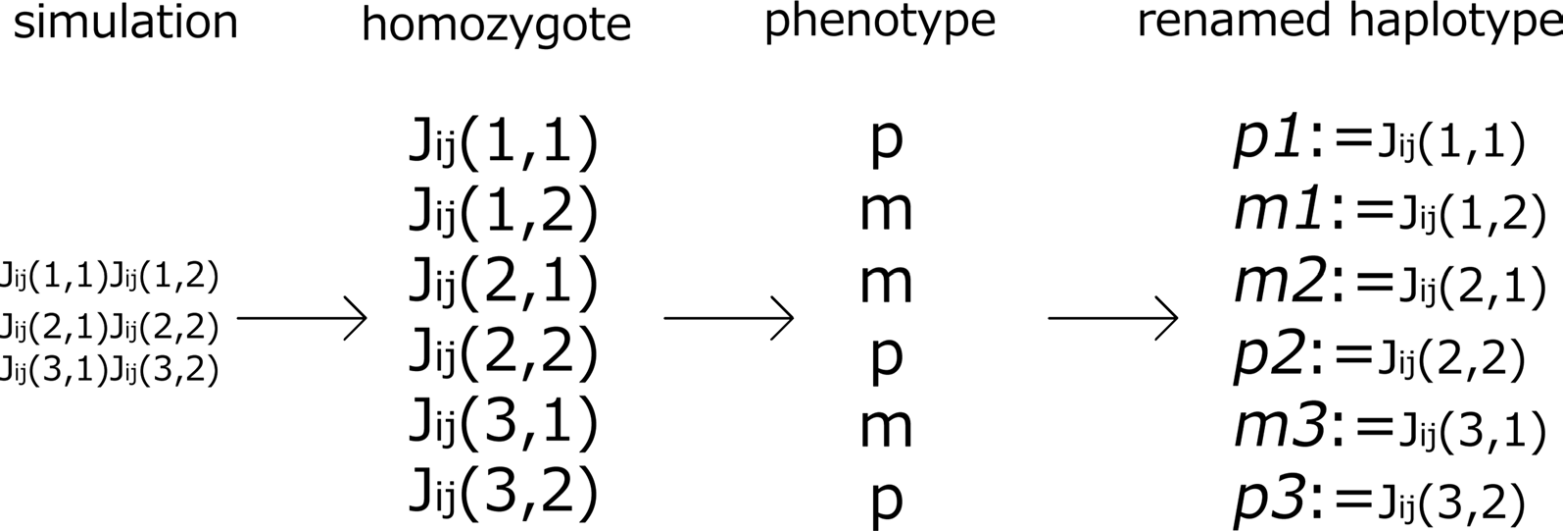
Renaming of haplotypes
3. Forming a pair of groups of genomes to test the “homozygote” and heterozygote cases (Fig. 11)

**Fig. 11:**
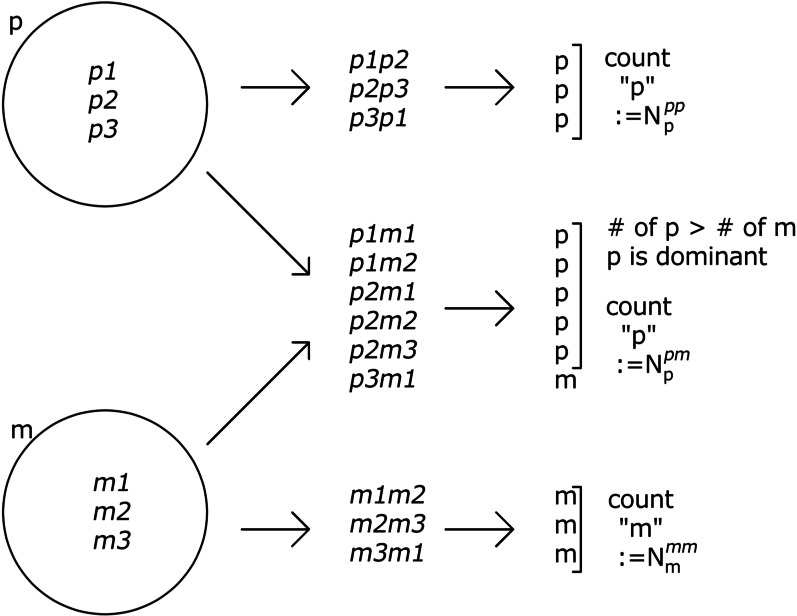
Testing the homozygote and heterozygote cases
  Genome groups are defined as a group of genomes that express the same phenotype (e.g., ${p_1}$, ${p_2}$, ...). To test Mendel’s law, we made two genome groups, p and m. From these, we made sufficient homozygote and heterozygote samples to test whether GMD was achieved.
  To test the homozygote case, $N_{samples}$ of homozygotes were generated from one genome group by choosing two haplotypes at random. Note that the homozygote here does not necessarily contain the same $J_{ij}$. Then, the phenotypes of the “homozygote” were computed. The number of samples for which the phenotypes were the same as the phenotype of the original genome group was counted. This number was defined as $N^{pp}_{p}$ and $N^{m m}_{m}$ for the phenotypes p and m, respectively.
  To test the heterozygote case, heterozygotes are made by two haplotypes chosen from different genome groups. We chose $2N_{samples}$ of the heterozygote to perform a rigorous comparison with classic Mendelian dominance. By computing the phenotype of each heterozygote, we obtained the number of dominant phenotypes, which was defined as $N^{pm}_{p}$ when *p* is dominant. Here, the samples producing the dominant phenotype were defined as the phenotype that frequently appears in the heterozygote result .
4. Calculating the GMR
  Following the GMD, the GMR was computed by
  5 $$\begin{aligned} H = \frac{(N^{pp}_{p}+N^{pm}_{p}+N^{m m}_{m})}{4N_{{sample}}}. \end{aligned}$$

When the GMR is 1, complete GMD is achieved. Conversely, when the GMR is 0.5, the phenotype is determined at random without any dominance.

Note that $N^{pm}_{p}$ is sampled in the doubled population $2N_{{sample}}$ so that the maximum value of $N^{pm}_{p}$ is also double the value of that of $N^{pp}_{p}$ or $N^{m m}_{m}$. Therefore, the maximum value of *H* is 1. When the dynamics produce a phenotype randomly, $N^{pp}_{p}=N^{mm}_{m}=0.5N_{{sample}}$ and $N^{pp}_ {p}=N_{ {sample}}$, so $H=0.5$. When the dynamics always produce the same phenotype, for instance p, $N^{pp}_ {p}=N_{{sample}}$, $N^{mm}_{m}=0$ and $N^{pp}_{m}=2N_{{sample}}$, so $H=0.75$. Hence, a GMR value beyond 0.75 indicates the emergence of GMD beyond the trivial case with just a single phenotype.

Note that the frequency of genes may be biased by following Hardy–Weinberg equilibrium. By using the above method of sampling, one can focus only on the phenotypic distribution, as Mendel originally did.

### Robustness against initial noise

We computed the SD of fitness over (20) different initial conditions by setting $\{x_i(0)\times (1-\eta (0,0.1))\}$, for a given individual chosen from an evolved population. We then computed the average of isogenic SD over 100 individuals in the evolved population. The averaged SD thus computed is displayed in Fig. 6.

### Distance between two genomes

To measure genetic variance, the distance between two genomes was calculated. The distance ($\sum ^N_{i=1}\sum ^N_{j=1}|J^{(1)}_{ij}-J^{(2)}_{ij}|$) was calculated for two genomes, $J^{(1)}_{ij}$ and $J^{(2)}_{ij}$, in the diploid cell. Then, the average distance over all individuals was computed.

## Supplementary Information

**Additional file 1: Fig. S1.** Two-genome distance as a function of mutation rate (including the recombination-only case). The distance was computed as the Euclidean distance between the matrix elements in two genomes, J^1^_ij_ and J^2^_ij_. We simulated GRNs with 20 genes, such that the number of matrix elements, that is, the maximal Hamming distance (i.e., the number of different elements), was 20 × 20 = 400. Consequently, the GMR (and the fitness) maintains a high value until half the elements have been changed. The value of the 10^4^-th generation is shown. Two-genome distances are shown for the RO case (orange triangle), asexual case (black dot), MO case (green diamond), and MR case (blue triangle). The noise amplitude was 10^4^. The RO case behaves along with the asexual case.

**Additional file 2: Fig. S2.** Increase of GMR along the evolution in the asymmetric dynamics case. Frequency of group Mendelian ratio in the 0th, 100th, and 1499th generation. Each focused gene calculates this frequency in 30 samples. GMR is increased along with the evolution, and over 10% of them reach complete group Mendelian dominance. Note that the distribution is shifted because of the asymmetry, and the peak of the distribution of GMR in the 0th generation reaches 0.75 even though their GRNs are random networks. This indicates that this asymmetry promotes GMD. The mutation rate per edge = 10^4^ and the strength of noise = 10^4^. This frequency is measured in the MR mode. The bin is 0.05.

## Data Availability

All data generated or analyzed during this study are included in this published article and its supplementary information files.

## Abbreviations

GRN: Gene Regulatory Network
MO: Meiosis Only
MR: Meiosis and Recombination
RO: Recombination Only
GMR: Group Mendelian Ratio
GMD: Group Mendelian Dominance
